# Supplementary material for: The Role of Statins on Helicobacter pylori Eradication: Results from the European Registry on the Management of H. pylori (Hp-EuReg)
Source: Antibiotics (Basel). 2021 Aug 11;10(8):965. doi: 10.3390/antibiotics10080965 (PMC8388917; doi:10.3390/antibiotics10080965)
Supplement: Supplementary file 1 [file antibiotics-10-00965-s001.zip › antibiotics-1297329-supplementary.pdf]

SUPPLEMENTARY MATERIAL

Supplementary file S1. Contribution log.

Manuel Castro-Fernández. Hospital de Valme, Sevilla, SPAIN. Acquired data, critically reviewed the manuscript drafts, and approved the submitted manuscript.

Manuel Pabón-Carrasco. Centro Universitario de Cruz Roja, Universidad de Sevilla, Sevilla, SPAIN. Collected and helped interpreting data, critically reviewed the manuscript drafts, and approved the submitted manuscript.

Ángel Cosme. Hospital Donostia/Instituto Biodonostia and CIBERehd, Universidad del País Vasco (UPV/EHU), San Sebastián, SPAIN. Collected and helped interpreting data, critically reviewed the manuscript drafts, and approved the submitted manuscript.

Ilaria Maria Saracino. S. Orsola Malpighi Hospital, Bologna, ITALY. Collected and helped interpreting data, critically reviewed the manuscript drafts, and approved the submitted manuscript.

Giulia Fiorini. S. Orsola Malpighi Hospital, Bologna, ITALY. Collected and helped interpreting data, critically reviewed the manuscript drafts, and approved the submitted manuscript.

Benito Velayos. Hospital Clínico Universitario de Valladolid, Valladolid, SPAIN. Acquired data, critically reviewed the manuscript drafts, and approved the submitted manuscript.

Noelia Alcaide. Hospital Clínico Universitario de Valladolid, Valladolid, SPAIN. Collected and helped interpreting data, critically reviewed the manuscript drafts, and approved the submitted manuscript.

Pedro Luis González Cordero. Mata-Romero. Hospital San Pedro de Alcántara and CIBERehd, Cáceres, SPAIN. Collected and helped interpreting data, critically reviewed the manuscript drafts, and approved the submitted manuscript.

Carmen Dueñas Sadornil. Hospital San Pedro de Alcántara and CIBERehd, Cáceres, SPAIN. Collected and helped interpreting data, critically reviewed the manuscript drafts, and approved the submitted manuscript.

Manuel Alfonso Jimenez Moreno. Complejo Asistencial Universitario de Burgos, Burgos, SPAIN. Acquired data, critically reviewed the manuscripts' drafts, and approved the submitted manuscript

Ángel Lanas. Hospital Clínico Universitario Lozano Blesa and CIBERehd, Zaragoza, SPAIN. Collected and helped interpreting data, critically reviewed the manuscript drafts, and approved the submitted manuscript.

Enrique Alfaro Almajano. Hospital Clínico Universitario Lozano Blesa and CIBERehd, Zaragoza, SPAIN. Collected and helped interpreting data, critically reviewed the manuscript drafts, and approved the submitted manuscript.

Luisa Carmen De La Peña Negro. Hospital de Viladecans, Barcelona, SPAIN. Collected and helped interpreting data, critically reviewed the manuscript drafts, and approved the submitted manuscript.

Pedro Delgado Guillena. Hospital de Mérida, Badajoz, SPAIN. Collected and helped interpreting data, critically reviewed the manuscript drafts, and approved the submitted manuscript.

Blas José Gómez-Rodríguez. Hospital Universitario Virgen Macarena, Sevilla, SPAIN. Collected and helped interpreting data, critically reviewed the manuscript drafts, and approved the submitted manuscript.

Elida Oblitas Susanibar. Consorci Sanitari de Terrassa, Barcelona, SPAIN. Collected and helped interpreting data, critically reviewed the manuscript drafts, and approved the submitted manuscript

Josep María Botargués. Hospital Universitari de Bellvitge, Barcelona, SPAIN. Collected and helped interpreting data, critically reviewed the manuscript drafts, and approved the submitted manuscript.

Sotirios D. Georgopoulos. Gastroenterology Department, Athens Medical, P. Faliron Hospital, Athens, GREECE. Collected and helped interpreting data, critically reviewed the manuscript drafts, and approved the submitted manuscript.

Frode Lerang. Gastroenterololgy, Østfold Hospital Trust, Grålum, NORWAY. Collected and helped interpreting data, critically reviewed the manuscript drafts, and approved the submitted manuscript.

Ian Beales. Medicine, University of East Anglia Norwich Medical School, Norwich NR4 7UY, Norfolk, UK. Collected and helped interpreting data, critically reviewed the manuscript drafts, and approved the submitted manuscript.

Mónica Perona. Hospital Quirón Marbella, Málaga, SPAIN. Collected and helped interpreting data, critically reviewed the manuscript drafts, and approved the submitted manuscript.

Alain Huerta. Hospital de Galdakao-Usansolo, Vizcaya, SPAIN. Collected and helped interpreting data, critically reviewed the manuscript drafts, and approved the submitted manuscript.

Teresa Angueira. Hospital General de Tomelloso and CIBERehd, Ciudad Real, SPAIN. Collected and helped interpreting data, critically reviewed the manuscript drafts, and approved the submitted manuscript.

Halis Simsek. Internal Medicine/Gastroenterology, Hacettepe University Faculty of Medicine, Ankara, TURKEY. Collected and helped interpreting data, critically reviewed the manuscript drafts, and approved the submitted manuscript.

Cem Simsek Internal. Medicine/Gastroenterology, Hacettepe University Faculty of Medicine, Ankara, TURKEY. Collected and helped interpreting data, critically reviewed the manuscript drafts, and approved the submitted manuscript.

Vendel A. Kristensen. Department of Medicine, Unger-Vetlesens Institute, Lovisenberg Diakonale Hospital, Oslo, NORWAY. Collected and helped interpreting data, critically reviewed the manuscript drafts, and approved the submitted manuscript.

Ana Campillo. Hospital Reina Sofía, Tudela, Navarra, SPAIN. Collected and helped interpreting data, critically reviewed the manuscript drafts, and approved the submitted manuscript.

Rosario Antón. Hospital Clínic Universitari de Vàlencia, Valencia, SPAIN. Collected and helped interpreting data, critically reviewed the manuscript drafts, and approved the submitted manuscript.

Natalia Nikolaevna Dekhnich. Institute of Antimicrobial Chemotherapy. Smolensk, RUSSIA. Collected and helped interpreting data, critically reviewed the manuscript drafts, and approved the submitted manuscript.

Bárbara Gómez. Hospital de Mataró, Barcelona, SPAIN. Collected and helped interpreting data, critically reviewed the manuscript drafts, and approved the submitted manuscript.

Marina F. Osipenko. Novosibirsk State Medical University FGBOU in the NSMU of the Ministry of Health of Russia, RUSSIA. Collected and helped interpreting data, critically reviewed the manuscript drafts, and approved the submitted manuscript.

Oleg Zaytsev. First Clinical Medical Centre, Kovrov, RUSSIA. Collected and helped interpreting data, critically reviewed the manuscript drafts, and approved the submitted manuscript.

Eduardo Iyo. Hospital Comarcal de Inca, Mallorca, SPAIN. Collected and helped interpreting data, critically reviewed the manuscript drafts, and approved the submitted manuscript.

Laimas Jonaitis. Lithuanian University of Health Sciences, Kaunas, LITHUANIA. Collected and helped interpreting data, critically reviewed the manuscript drafts, and approved the submitted manuscript.

Doron Boltin. Division of Gastroenterology, Rabin Medical Center, Petah Tikva, and the Sackler School of Medicine, Tel Aviv University, Tel Aviv, ISRAEL. Collected and helped interpreting data, critically reviewed the manuscript drafts, and approved the submitted manuscript.

Dragan Jurcic. Department of Gastroenterology, Internal Medicine Clinic, Clinical Hospital Sveti Duh, Sveti Duh, Zagreb, CROATIA. Collected and helped interpreting data, critically reviewed the manuscript drafts, and approved the submitted manuscript.

Marinko Marusic. Department of Gastroenterology, Internal Medicine Clinic, Clinical Hospital Sveti Duh, Sveti Duh, Zagreb, CROATIA. Collected and helped interpreting data, critically reviewed the manuscript drafts, and approved the submitted manuscript.

Ramón Pajares-Villaroya. Hospital Infanta Sofía, Madrid, SPAIN. Collected and helped interpreting data, critically reviewed the manuscript drafts, and approved the submitted manuscript.

Roald Torp. Department of Gastroenterology, Hamar Hospital, Hamar, NORWAY. Collected and helped interpreting data, critically reviewed the manuscript drafts, and approved the submitted manuscript.

Jose Luis Dominguez-Jiménez. Hospital Alto del Guadalquivir, Jaén, SPAIN. Collected and helped interpreting data, critically reviewed the manuscript drafts, and approved the submitted manuscript.

Peter Malfertheiner. Department of Gastroenterology, Hepatology and Infectious Diseases, Otto-von-Guericke University of Magdeburg, Magdeburg, GERMANY. Collected and helped interpreting data, critically reviewed the manuscript drafts, and approved the submitted manuscript.

Marino Venerito. Department of Gastroenterology, Hepatology and Infectious Diseases, Otto-von-Guericke University of Magdeburg, Magdeburg, GERMANY. Collected and helped interpreting data, critically reviewed the manuscript drafts, and approved the submitted manuscript.

Rosa Rosania. Department of Gastroenterology, Hepatology and Infectious Diseases, Otto-von-Guericke University of Magdeburg, Magdeburg, GERMANY. Collected and helped interpreting data, critically reviewed the manuscript drafts, and approved the submitted manuscript.

Marianela Mego. Hospital Universitario General de Catalunya, Barcelona, SPAIN. Collected and helped interpreting data, critically reviewed the manuscript drafts, and approved the submitted manuscript.

Irina Voynovan. Digestive Unit, Moscow Clinical Scientific Center Named After A.S. Loginov, Moscow, RUSSIA. Collected and helped interpreting data, critically reviewed the manuscript drafts, and approved the submitted manuscript.

Edurne Amorena. Complejo Hospitalario de Navarra, Pamplona, SPAIN. Collected and helped interpreting data, critically reviewed the manuscript drafts, and approved the submitted manuscript.

Javier Alcedo. Hospital de Barbastro, Huesca, SPAIN. Collected and helped interpreting data, critically reviewed the manuscript drafts, and approved the submitted manuscript.

Natalia V. Bakulina. Department of Therapy and Clinical Pharmacology, North-Western State Medical University, Sankt-Peterburg, RUSSIA. Collected and helped interpreting data, critically reviewed the manuscript drafts, and approved the submitted manuscript.

Igor Bakulin. Department of Therapy and Clinical Pharmacology, North-Western State Medical University, Sankt-Peterburg, RUSSIA. Collected and helped interpreting data, critically reviewed the manuscript drafts, and approved the submitted manuscript.

Neven Ljubicic. Division of Gastroenterology, Department of Internal Medicine, Sestre milosrdnice University Hospital Center, School of Medicine and Dental Medicine, University of Zagreb, Zagreb, CROATIA. Collected and helped interpreting data, critically reviewed the manuscript drafts, and approved the submitted manuscript.

Marko Nikolic. Division of Gastroenterology, Department of Internal Medicine, Sestre milosrdnice University Hospital Center, School of Medicine and Dental Medicine, University of Zagreb, Zagreb, CROATIA. Collected and helped interpreting data, critically reviewed the manuscript drafts, and approved the submitted manuscript.

Marco Romano. Dipartimento di "Medicina di Precisione", UOC Epatogastroenterologia, Università della Campania "Luigi Vanvitelli", Napoli, ITALY. Collected and helped interpreting data, critically reviewed the manuscript drafts, and approved the submitted manuscript.

Antonietta G. Gravina. Dipartimento di "Medicina di Precisione", UOC Epatogastroenterologia, Università della Campania "Luigi Vanvitelli", Napoli, ITALY. Collected and helped interpreting data, critically reviewed the manuscript drafts, and approved the submitted manuscript.

Adi Lahat. Department of Gastroenterology, Sheba Medical Center, Tel Hashomer, affiliated with Sackler School of Medicine, Tel Aviv University, Ramat Gan, ISRAEL. Collected and helped interpreting data, critically reviewed the manuscript drafts, and approved the submitted manuscript.

Pilar Sánchez-Pobre. Dirección General del Proceso Integrado de Salud, Madrid, SPAIN. Collected and helped interpreting data, critically reviewed the manuscript drafts, and approved the submitted manuscript.

Dan L. Dumitrascu. 2nd Dept. of Internal Medicine, 'Iuliu Hatieganu' University of Medicine and Farmacy, Cluj-Napoca, ROMANIA. Collected and helped interpreting data, critically reviewed the manuscript drafts, and approved the submitted manuscript.

Mila Kovacheva-Slavova. Department of Gastroenterology, Queen Yoanna University Hospital, Medical University of Sofia, Sofia, Bulgaria. Collected and helped interpreting data, critically reviewed the manuscript drafts, and approved the submitted manuscript.

Albert Tomàs. Consorci Sanitari del Garraf, Sant Pere de Ribes, Barcelona, SPAIN. Collected and helped interpreting data, critically reviewed the manuscript drafts, and approved the submitted manuscript.

Gyorgy M. Buzás. Department of Gastroenterology, Ferencváros Health Center, Budapest, HUNGARY. Collected and helped interpreting data, critically reviewed the manuscript drafts, and approved the submitted manuscript.

Miguel Fernández-Bermejo. Clínica San Francisco, Cáceres, SPAIN. Collected and helped interpreting data, critically reviewed the manuscript drafts, and approved the submitted manuscript.

Tatiana Ilchishina. SM-Clinic, Saint-Petersburg, RUSSIA. Collected and helped interpreting data, critically reviewed the manuscript drafts, and approved the submitted manuscript.

Maja Seruga. General Hospital Murska Sobota, Murska Sobota, SLOVENIA. Collected and helped interpreting data, critically reviewed the manuscript drafts, and approved the submitted manuscript.

Xavier Calvet. Corporació Sanitària Universitària Parc Taulí. CIBERehd, Instituto de Salud Carlos III Departament de Medicina. Universitat Autònoma de Barcelona, Sabadell, SPAIN. Collected and helped interpreting data, critically reviewed the manuscript drafts, and approved the submitted manuscript.

Ekaterina Y. Plotnikova. Kemerovo State Medical Academy of the Ministry of Health of the Russian Federation, RUSSIA. Collected and helped interpreting data, critically reviewed the manuscript drafts, and approved the submitted manuscript.

Zaza Beniashvili. Division of Gastroenterology, Rabin Medical Center (Sharon Campus), Petah Tikva, ISRAEL. Collected and helped interpreting data, critically reviewed the manuscript drafts, and approved the submitted manuscript.

Ana Garre. Hospital Universitario de La Princesa, Instituto de Investigación Sanitaria Princesa (IIS-IP), Universidad Autónoma de Madrid (UAM) and Centro de Investigación Biomédica en Red de Enfermedades Hepáticas y Digestivas (CIBERehd), Madrid, SPAIN, Collected and helped interpreting data, critically reviewed the manuscript drafts, and approved the submitted manuscript.

Alicia C. Marín. Hospital Universitario de La Princesa, Instituto de Investigación Sanitaria Princesa (IIS-IP), Universidad Autónoma de Madrid (UAM) and Centro de Investigación Biomédica en Red de Enfermedades Hepáticas y Digestivas (CIBERehd), Madrid, SPAIN. Collected and helped interpreting data, critically reviewed the manuscript drafts, and approved the submitted manuscript.

Almudena Durán. Hospital Universitario de La Princesa, Instituto de Investigación Sanitaria Princesa (IIS-IP), Universidad Autónoma de Madrid (UAM) and Centro de Investigación Biomédica en Red de Enfermedades Hepáticas y Digestivas (CIBERehd), Madrid, SPAIN. Collected and helped interpreting data, critically reviewed the manuscript drafts, and approved the submitted manuscript.

Jennifer Fernandez Pacheco. Hospital Universitario de La Princesa, Instituto de Investigación Sanitaria Princesa (IIS-IP), Universidad Autónoma de Madrid (UAM) and Centro de Investigación Biomédica en Red de Enfermedades Hepáticas y Digestivas (CIBERehd), Madrid, SPAIN. Collected and helped interpreting data, critically reviewed the manuscript drafts, and approved the submitted manuscript.

Supplementary file S2: Proton pump inhibitor (PPI) categories: low, standard and high acid inhibition [1, 2].

Low dose PPI: ranging from 4.5 to 27 mg omeprazole equivalents, b.i.d. (i.e. 20 mg omeprazole equivalents, b.i.d.).

Standard dose PPI: ranging from 32 to 40 mg omeprazole equivalents, b.i.d. (i.e. 40 mg omeprazole equivalents, b.i.d.).

High dose PPI: ranging from 54 to 128 mg omeprazole equivalents, b.i.d. (i.e. 60 mg omeprazole equivalents, b.i.d.).

1. Kirchheiner J, Glatt S, Fuhr U, et al. Relative potency of proton-pump inhibitors-comparison of effects on intragastric pH. Eur J Clin Pharmacol 2009;65(1):19-31.
2. Graham DY, Lu H, Dore MP. Relative potency of proton-pump inhibitors, Helicobacter pylori therapy cure rates, and meaning of double-dose PPI. Helicobacter 2019;24(1):e12554.

Table S1. Inclusion of patients per country.

Country	Frequency (N)	Percentage (%)
**Spain**	6313	59
**Slovenia**	1268	12
**Italy**	602	5.6
**Russia**	595	5.6
**Ukraine**	533	5
**Lithuania**	225	2.1
**Portugal**	184	1.7
**Greece**	178	1.7
**Latvia**	162	1.5
**United kingdom**	152	1.4
**Norway**	136	1.3
**Israel**	75	0.7
**Croatia**	51	0.5
**Turkey**	49	0.5
**Romania**	47	0.4
**France**	31	0.3
**Hungary**	25	0.2
**Germany**	21	0.2
**Bulgary**	17	0.2
**Ireland**	10	0.1
**Belgium**	5	0.0
**Denmark**	4	0.0
**Serbia**	4	0.0
**Switzerland**	3	0.0
**Czech republic**	2	0.0
**Total**	10,692	100

N: total number of patients included.

Table S2. Distribution of eradication therapies prescribed according to the statin used.

			**Simvastatin N (%)**	**Atorvastatin N (%)**	**Rosuvastatin N (%)**	**Others N (%)**
Empirical prescription	First-line	N included	101 (100)	83 (100)	28 (100)	12 (100)
		PPI+C+A	42 (42)	36 (43)	10 (36)	4
		PPI+C+M/T	11 (11)	9 (11)	5 (18)	2
		PPI+M/T+A	--	1 (1)	--	--
		PPi+Mx+A	1 (1)	--	--	--
		PPI+C+A+M/T (seq)	7 (7)	5 (6)	3 (11)	3
		PPI+C+A+M/T (conc)	3 (33)	20 (24)	6 (21)	3
		PPI+L+A	1 (1)	1 (1)	1 (4)	--
		PPI+C+A+B	2 (2)	5 (6)		--
		PPI + single capsule*	2 (2)	6 (7)	2 (7)	--
		PPI+M/T+Tc+B	1 (1)	--	--	--
		PPI+L+A+B	1 (1)	--	--	--
		PPI+A	--	--	1 (4)	--
	Rescue lines	N included	34 (100)	23 (100)	7 (100)	3 (100)
		PPI+C+A	1 (3)	--	--	--
		PPI+C+M/T	1 (3)	--	--	--
		PPI+L+A	23 (68)	13 (57)	3 (43)	1 (33)
		PPi+Mx+A	1 (3)	1 (4)	--	1 (33)
		PPI+RF+A	--	1 (4)	--	1 (33)
		PPI+M/T+A	1 (3)	--	--	--
		PPI+C+A+M/T (seq)	1 (3)	--	--	--
		PPI+C+A+M/T (conc)	1 (3)	2 (9)	1 (14)	--
		PPI+C+A+B	--	1 (4)	--	--
		PPI+L+A+B	1 (3)	--	--	--
		PPI + single capsule*	--	2 (9)	1 (14)	--
		PPI+M/T+Tc+B	2 (6)	2 (9)	--	--
		PPI+M/T+D+B	1 (3)	--	--	--
		Other	1 (3)	1 (4)	2 (28)	---
Culture-guided prescription	First-line	N included	19 (100)	24 (100)	2 (100)	2 (100)
		PPI+C+A	14 (74)	16 (67)	2 (100)	2 (100)
		PPI+M/T+A	--	1 (4)		
		PPI+RF+A	--	1 (4)	--	--
		PPI+C+A+M/T (seq)	1 (5)	--	--	--
		PPI+C+A+M/T (conc)	1 (5)	4 (17)	--	--
		PPI+C+A+B	1 (5)	2 (8)	--	--
		PPI + single capsule*	2 (2)	--	--	--
		Other	2 (10)	--	--	--
	Rescue lines	N included	1 (100)	2 (100)	--	--
		PPI+RF+A	--	1 (50)	--	--
		Other	1 (100)	1 (50)	--	--

N: total number of patients included. %: proportion of patients receiving the eradication treatment according to the statin use. Others: includes pravastatin, pitavastatin, lovastatin or fluvastatin. PPI: proton pump inhibitor. C: clarithromycin. A: amoxicillin. M: metronidazole. T: tinidazole. Mx: moxifloxacin. Seq: sequential administration. Conc: concomitant administration. L: levofloxacin. B: bismuth. Single capsule*: three-in-one single capsule containing bismuth, tetracycline and metronidazole. Tc: tetracycline. RF: rifabutin. D. doxycycline. Others: other minority therapies. --: No patients available.

Table S3. Adverse events reported in each group according to severity.

AE	Empirical Prescription				Culture-Guided Prescription			
	First-line		Rescue-line		First-line		Rescue-line	
	Statin-users N (%)	Non-statin users N (%)	Statins-users	Non-statin users N (%)	Statin-users N (%)	Non-statin users N (%)	Statin-users N (%)	Non-statin users N (%)
Total N	361	1,164	199	438	26	81	8	20
**Dysgeusia**	112	477	36	104	7	28	2	2
Mild	81 (72)	295 (62)	23 (64)	61 (59)	5 (72)	11 (39)	1 (50)	2 (100)
Moderate	28 (25)	160 (33)	12 (33)	37 (35)	1 (14)	16 (57)	1 (50)	0
Severe	3 (3)	22 (5)	1 (3)	6 (6)	1 (14)	1 (4)	0	0
**Diarrhea**	126	357	108	187	12	21	0	8
Mild	71 (56)	232 (65)	29 (27)	69 (37)	8 (67)	9 (43)	0	5 (63)
Moderate	51 (41)	115 (32)	79 (73)	111 (59)	4 (33)	11 (52)	0	0
Severe	4 (3)	10 (3)	0	7 (4)	0	1 (5)	0	3 (37)
**Nausea**	114	395	83	178	6	20	4	5
Mild	61 (53)	247 (63)	40 (48)	84 (47)	5 (83)	13 (65)	3 (75)	3 (60)
Moderate	50 (44)	123 (31)	42 (51)	89 (50)	1 (17)	6 (30)	1 (25)	2 (40)
Severe	3 (3)	25 (6)	1 (1)	5 (3)	0	1 (5)	0	0
**Vomits**	40	136	44	99	1	6	0	1
Mild	19 (47)	86 (63)	27 (61)	58 (59)	1 (100)	5 (83)	0	1 (100)
Moderate	21 (53)	37 (27)	17 (39)	38 (38)	0	1 (17)	0	0
Severe	0	13 (10)	0	3 (3)	0	0	0	0
**Dyspepsia**	39	141	35	77	2	11	1	1
Mild	21 (54)	63 (45)	14 (40)	25 (33)	2 (100)	6 (55)	0	1 (100)
Moderate	15 (38)	68 (48)	21 (60)	51 (66)	0	4 (36)	1 (100)	0
Severe	3 (8)	10 (7)	0	1 (1)	0	1 (9)	0	0
**Heartburn**	18	45	17	22	0	4	0	1
Mild	11 (61)	14 (31)	1 (6)	3 (14)	0	2 (50	0	1 (100)
Moderate	7 (39)	24 (53)	13 (76)	19 (86)	0	2 (50)	0	0
Severe	0	7 (16)	3 (18)	0	0	0	0	0
**Abdominal pain**	50	196	24	57	2	17	1	3
Mild	29 (58)	132 (67)	14 (58)	32 (56)	1 (50)	13 (76)	1 (100)	0
Moderate	18 (36)	48 (25)	10 (42)	21 (37)	1 (50)	3 (18)	0	1 (33)
Severe	3 (6)	16 (8)	0	4 (7)	0	1 (6)	0	2 (67)
**Asthenia**	52	155	41	66	3	7	0	3
Mild	23 (44)	66 (42)	8 (19)	24 (36)	3 (100)	3 (43)	0	1 (33)
Moderate	27 (52)	71 (46)	27 (66)	39 (59)	0	4 (57)	0	2 (67)
Severe	2 (4)	18 (12)	6 (15)	3 (5)	0	0	0	0
**Anorexia**	28	88	55	72	1	6	2	4
Mild	9 (32)	25 (28)	10 (18)	9 (12)	1 (100)	2 (33)	1 (50)	2 (50)
Moderate	16 (57)	55 (63)	39 (71)	56 (78)	0	2 (33)	1 (50)	2 (50)
Severe	3 (11)	8 (9)	6 (11)	7 (10)	0	2 (33)	0	0

Rescue-lines: embracing therapies prescribed from 2nd-6th eradication attempt. AE: adverse events. N: total number of patients included. %: proportion of patients included.

Table S4. Effectiveness and safety in empirical therapies according to the type of statin used.

				Simvastatin	Atorvastatin	Rosuvastatin	Others	p-value
First-line (N =224)	Overall	mITT E	N (%)	83 (87)	69 (92)	22 (81.5)	11 (100)	0.278
			95% CI	79-93	83-97	62-94	NA	
		AE	N (%)	15 (15)	10 (12)	5 (18)	2 (18)	0.876
			95% CI	9-23	6-22	6-37	2-52	
	PPI+C+A	mITT E	N (%)	33 (82.5)	26 (90)	7 (70)	3 (100)	0.376
			95% CI	67-93	73-98	35-93	NA	
		AE	N (%)	3 (7)	3 (9)	2 (20)	0 (0)	0.595
			95% CI	2-19	2-23	3-56	--	
	PPI+C+A+M/T (conc)	mITT E	N (%)	30 (97)	19 (95)	5 (100)	3 (100)	0.875
			95% CI	83-100	75-100	NA	NA	
		AE	N (%)	8 (24)	3 (15)	1 (17)	1 (33)	0.804
			95% CI	11-42	3-38	1-64	1-91	
	PPI+C+M/T	mITT E	N (%)	10 (91)	7 (88)	5 (100)	2 (100)	0.712
			95% CI	59-100	47-100	NA	NA	
		AE	N (%)	3 (27)	2 (25)	2 (40)	1 (50)	0.872
			95% CI	6-61	3-65	5-85	1-99	
	PPI+C+A+M/T (seq)	mITT E	N (%)	5 (83)	5 (100)	3 (100)	3 (100)	0.532
			95% CI	36-100	NA	NA	NA	
		AE	N (%)	0 (0)	0 (0)	0 (0)	0 (0)	NA
			95% CI	--	--	--	--	
	PPI+ single-capsule*	mITT E	N (%)	2 (100)	6 (100)	1 (50)	NA	0.155
			95% CI	NA	NA	*1-99	NA	
		AE	N (%)	0 (0)	0 (0)	0 (0)	NA	NA
			95% CI	--	--	--	NA	
Rescue lines (N = 67)	Overall	mITT E	N (%)	23 (72)	20 (87)	5 (71)	3 (100)	0.318
			95% CI	53-86	66-97	29-96	NA	
		AE	N (%)	3 (9)	6 (26)	0 (0)	0 (0)	0.104
			95% CI	2-24	10-48	--	--	
	PPI+L+A	mITT E	N (%)	15 (71)	11 (85)	1 (33)	1 (100)	0.297
			95% CI	48-89	55-98	1.91	NA	
		AE	N (%)	3 (13)	3 (23)	0 (0)	0 (0)	0.581
			95% CI	3-34	5-54	--	--	

Only therapies administered to at least 10 patients with the exact statin specified are included. mITT E: modified intention-to-treat effectiveness. AE: adverse events. N: total number of patients included. %: proportion of patients presenting with effectiveness or the adverse event. CI: confidence interval. PPI: proton pump inhibitor. C: clarithromycin. A: amoxicillin. M: metronidazole. T: tinidazole. Single capsule*: three-in-one single capsule containing bismuth, tetracycline and metronidazole. Conc: concomitant administration. Seq: sequential administration. L: levofloxacin. NA: not applicable. --: No patients available in analysis.

Table S5. Effectiveness and safety in culture-guided therapies according to the type of statin used.

				Simvastatin	Atorvastatin	Rosuvastatin	Others	p-value
First-line (N = 47)	Overall	mITT E	N (%)	15 (88)	22 (96)	2 (100)	2 (100)	0.714
			95% CI	64-99	78-100	NA	NA	
		AE	N (%)	0 (0)	4 (17)	0 (0)	0 (0)	0.125
			95% CI	--	5-39	--	--	
	PPI+C+A	mITT E	N (%)	12 (100)	14 (93)	2 (100)	2 (100)	0.685
			95% CI	NA	68-100	--	--	
		AE	N (%)	0 (0)	2 (13)	0 (0)	0 (0)	0.364
			95% CI	--	2-41	--	--	
Rescue lines (N = 3)	Overall	mITT E	N (%)	1 (100)	1 (100)	NA	NA	NA
			95% CI	NA	NA	NA	NA	
		AE	N (%)	0 (0)	1 (100)	NA	NA	1
			95% CI	--	NA	NA	NA	

Only therapies administered to at least 10 patients with the exact statin specified are included. mITT E: modified intention-to-treat effectiveness. AE: adverse events. N: total number of patients included. %: proportion of patients presenting with effectiveness or the adverse event. CI: confidence interval. PPI: proton pump inhibitor. C: clarithromycin. A: amoxicillin. NA: not applicable. --: No patients available in analysis.
